# Similarity-Based Method with Multiple-Feature Sampling for Predicting Drug Side Effects

**DOI:** 10.1155/2022/9547317

**Published:** 2022-04-01

**Authors:** Zixin Wu, Lei Chen

**Affiliations:** College of Information Engineering, Shanghai Maritime University, Shanghai 201306, China

## Abstract

Drugs can treat different diseases but also bring side effects. Undetected and unaccepted side effects for approved drugs can greatly harm the human body and bring huge risks for pharmaceutical companies. Traditional experimental methods used to determine the side effects have several drawbacks, such as low efficiency and high cost. One alternative to achieve this purpose is to design computational methods. Previous studies modeled a binary classification problem by pairing drugs and side effects; however, their classifiers can only extract one feature from each type of drug association. The present work proposed a novel multiple-feature sampling scheme that can extract several features from one type of drug association. Thirteen classification algorithms were employed to construct classifiers with features yielded by such scheme. Their performance was greatly improved compared with that of the classifiers that use the features yielded by the original scheme. Best performance was observed for the classifier based on random forest with MCC of 0.8661, AUROC of 0.969, and AUPR of 0.977. Finally, one key parameter in the multiple-feature sampling scheme was analyzed.

## 1. Introduction

Drugs are important in treating various diseases; however, their therapeutic effects are accompanied by negative effects called side effects. In the pharmaceutical field, drug side effect is classified as an adverse drug reaction (ADR), the harmful or accidental reactions of qualified drugs that are irrelevant to the purpose of their use under normal usage and dosage. Some market-approved drugs may generate unaccepted side effects that can be harmful to the human body and bring high risks to pharmaceutical companies. For example, fluconazole and atorvastatin have potential hepatotoxicity and nephrotoxicity that can increase transaminase when used in specific patients such as those with liver disease. Side effects are one of the major obstacles in launching new drugs and delaying their development. Thus, determining all the side effects for a given drug is an important topic in drug development. Despite their efficiency in identifying side effects, solid clinical trials are time consuming and expensive and thus cannot meet the demand of large-scale tests. Thus, rapid and cheap methods for the identification of drug side effects must be developed.

Many advanced computational algorithms have been proposed [1–5] to provide strong technique support to deal with various medical problems. Several computational methods have been developed for the identification of drug side effects. Most of them are machine learning-based techniques that deeply investigate current information on drug side effects and develop proper patterns that can be used to predict side effects for a given new drug. Some early methods consisted of an individual binary classifier for each side effect [6–10]; hence, they always contain several binary classifiers that must be simultaneously executed to determine all side effects for a given drug. In view of this situation, some other techniques were directly built with multilabel classifiers [11–16] that identify side effects as labels and drugs as samples. Recommender systems were also proposed to predict drug side effects [17–19]. Recent works paired drugs and side effects as samples to convert the original problem as binary classification [20–22]. A key step in developing such binary classifiers is to extract essential properties from each drug–side effect pair. Some researchers used a similarity-based scheme to extract features [21, 22]; for convenience, they extracted only one feature from one type of drug association, a process called single-feature sampling scheme. However, some essential information may be omitted. For research continuation, a novel feature extraction scheme that can hold essential information for each drug–side effect pair must be developed.

In this study, an efficient binary classifier was proposed for the identification of drug side effects. Drugs and side effects were also paired as samples [20–22]. The single-feature sampling scheme [21, 22] was generalized to extract essential features from each pair. Named as multiple-feature sampling scheme, this newly proposed strategy can generate multiple features from each type of drug association. Classic machine learning algorithm, random forest (RF) [23], was adopted as the prediction engine. According to the 10-fold cross-validation results, the performance of such classifier was better than that of the previous classifier that uses original single sampling scheme for feature extraction. Further tests suggested that classifiers with other classification algorithms and features yielded by the multiple sampling scheme were all superior to those with the same classification algorithm and features generated by the original scheme. This finding indicated the power of the features generated by the proposed feature extraction scheme.

## 2. Materials and Methods

### 2.1. Benchmark Dataset

Data on 841 drugs and their side effects (824) [20–22] were extracted from SIDER (http://sideeffects.embl.de/) [24], a public database collecting the information of marketed drugs and their ADRs. The original data contained 888 drugs and 1385 side effects. The side effects that were annotated to no more than five drugs were excluded. Furthermore, drugs without the properties mentioned in Section 2.2 were discarded. From the remaining 841 drugs and 824 side effects, 57,058 drug–side effect pairs were obtained. Each pair indicated that the specific drug in the pair has the side effect in the same pair. Given that these pairs indicate the relationship between one drug and one side effect, they were termed as positive samples and comprised the positive dataset (PDS).

In addition to PDS, a negative dataset (NDS) was necessary in building an efficient binary classifier. A total of 57,058 drug–side effect pairs were produced by randomly pairing one drug and one side effect [20, 21]. However, no pairs can be labeled as positive samples. Therefore, these pairs constituted one NDS. Different NDSs may influence the performance of the classifier. Therefore, four other NDSs were also generated. Finally, five datasets each containing the PDS and one NDS were produced and denoted by *DS*_1_, *DS*_2_, *DS*_3_, *DS*_4_, and *DS*_5_.

### 2.2. Drug Association Obtained from Different Drug Properties

Two drugs with strong associations always share similar functions [25–29]. Side effects can be deemed as one type of drug function. Thus, classifiers can be constructed by adopting features derived from drug associations. From different aspects of drugs, several types of drug associations can be measured and quantified. For easy comparisons, the drug associations adopted in a previous study [21] were adopted, and their brief descriptions are as follows.

#### 2.2.1. Drug Fingerprint Association

Simplified molecular input line entry specification (SMILES) string [30] is a widely used scheme for drug representation. Fingerprints can be extracted from this string using existing software, such as RDKit [31]. The associations of two drugs can be evaluated by comparing their fingerprints. Here, ECFP_4 fingerprints and Tanimoto coefficient were used to measure such association between any two drugs. For formulation, this association for drugs *d*_1_ and *d*_2_ was denoted by *G*^*f*^(*d*_1_, *d*_2_).

#### 2.2.2. Drug Structural Association

In addition to SMILES string, another popular drug representation scheme is graph-based method. Here, each drug is represented by a graph with nodes depicting atoms and edges indicating bonds. The association of two drugs can be assessed by considering the similarity of two corresponding graphs. “SIMCOMP” (https://www.genome.jp/tools/simcomp/) reported in the KEGG [32, 33] was set up based on such idea. This tool can output the associations of a given drug with other drugs as measured by scores between 0 and 1. Such association for drugs *d*_1_ and *d*_2_ was denoted by *G*^*s*^(*d*_1_, *d*_2_).

#### 2.2.3. Drug Anatomical Therapeutic Chemical (ATC) Code Association

The ATC system is a widely accepted and used in drug classification. Each drug in such system is assigned five-level ATC codes that indicate its essential properties. For two drugs, their association can be measured according to their ATC codes. This study used the same method in [21] to evaluate drug association based on their ATC codes. For convenience, the association of drugs *d*_1_ and *d*_2_ was denoted by *G*^*a*^(*d*_1_, *d*_2_).

#### 2.2.4. Drug Literature Association

Given the extensive literature on drugs, the association of two drugs can be measured from their cooccurrence in some literature and natural language processing methods. The well-known public database, STITCH (version 4.0, http://stitch4.embl.de/) [34], provides such associations, which were directly employed in this study. “Textmining” score was extracted from the downloaded file “chemical_chemical.links.detailed.v4.0.tsv.” For drugs *d*_1_ and *d*_2_, their literature association was denoted by *G*^*tm*^(*d*_1_, *d*_2_).

#### 2.2.5. Drug Target Protein Association

Target protein is the basic property of drugs. Hence, the association of two drugs can be estimated by comparing their target proteins. In this study, the target proteins of drugs were retrieved from DrugBank (https://go.drugbank.com/) [35]. Each drug was encoded into a binary vector by applying one-hot scheme to its target proteins. The direction cosine of two vectors was defined as such association of two drugs. For formulation, this association between drugs *d*_1_ and *d*_2_ was denoted as *G*^*t*^(*d*_1_, *d*_2_).

### 2.3. Feature Engineering

**Figure 1:**
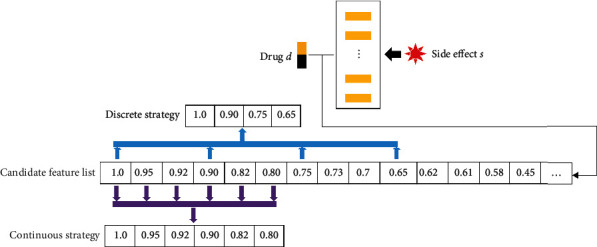
Procedures of multiple-feature sampling scheme to extract essential features from a drug–side effect pair. For a pair of drug *d* and side effect *s*, drugs having the side effect *s* are extracted from the training dataset. The association scores between *d* and these drugs constitute a candidate feature list. The discrete strategy selects discrete values in such list as features, and the continuous strategy picks up some top values in this list as features.

In Section 2.2, five types of drug associations that have been used to extract features to represent drug–side effect pairs [21, 22] were employed. These features indicated the linkage between one drug and one side effect in a drug–side effect pair. However, they extract only one feature from each type of drug association and thus cannot fully capture the essential linkage between the drug and the side effect. This study proposed a novel feature extraction scheme called multiple-feature sampling scheme, which can extract multiple features from one type of drug association. For a clear description, some denotations are necessary. For one drug–side effect pair *p* = <*d*, *s*>, where *d* and *s* indicate one drug and one side effect, respectively, let *S* be a set consisting of drugs having side effect *s* that have been extracted from the training dataset. If *d* also has side effect *s*, then, it would not be included in *S*. For one type of drug association, all values between *d* and drugs in *S* are selected. Denoted by Ψ^*k*^(*p*) (where *k* ∈ {*f*, *s*, *a*, *tm*, *t*} represents the type of drug association used to construct such list), a candidate feature list for *p* is then constructed with the decreasing order of above values. The top value in this list has been previously chosen as exclusive feature [21, 22]. Selection of several values in this list can contain more information to represent the linkage of drug *d* and side effect *s*. On the basis of the different selection models, two strategies were proposed, namely, discrete and continuous strategies. Their procedures are shown in Figure 1.

#### 2.3.1. Discrete Strategy

In this strategy, several values from the list Ψ^*k*^(*p*) are selected to indicate the distribution of values in the list. In this way, these selected values can fully indicate the linkage between drug *d* and side effect *s*. This process can be achieved by selecting some discrete values in the list. For example, the value at the first place or that at the top *q*% place can be selected. These values comprise a set of features from one type of drug association.

#### 2.3.2. Continuous Strategy

This strategy differs from the first one. Given that the linkage of drug *d* and side effect *s* is highly indicated by some top values in the list, these values must be properly selected because they may fully contain the essential information. For an integer *q* between 1 and 100, the top *q*% values in the list Ψ^*k*^(*p*) were selected as features.

### 2.4. Classification Algorithm

A proper classification algorithm is important in building an efficient classifier. In this study, RF [23] was adopted to construct the classifier. RF is one of the most classic classification algorithms and has been used to set up many classifiers in bioinformatics [36–41].

RF is an integrated classification algorithm containing several decision trees, each of which is constructed by two random selection procedures. The first procedure is to select samples. Given a dataset with *n* samples, randomly select *n* samples with replacement from such dataset. The second procedure is to select features to split each node. The selected features should be much less than overall features. After the predefined number of decision trees has been constructed, RF integrates them by major voting. For a query sample, each decision tree gives its prediction. The majority prediction is the predicted result of RF. Although a decision tree is a relative weak classification algorithm, RF is extremely powerful and has always been an important candidate to build different classifiers.

In this study, “RandomForest” in Weka [42] was directly used to implement the abovementioned RF. Default parameters were adopted, and the number of decision trees was set to 100.

In addition to RF, the following classification algorithms were used to build corresponding classifiers: support vector machine (SVM) (polynomial kernel, RBF kernel) [43], Adaboost M1 [44], Bagging [45], Bayesian network [46], Naive Bayes [47], *K*-nearest neighbor (KNN) [48], decision tree (C4.5) [49], PART [50], logistic regression [51], multilayer perceptron (MLP) [52], and Repeated Incremental Pruning to Produce Error Reduction (RIPPER) [53]. The goal is to confirm that the features yielded by the multiple sampling scheme are more effective than those yielded by the single sampling scheme. For convenience, corresponding tools in Weka were used to implement the above classification algorithms under default parameters. These classification algorithms adopt different principles and procedures for classification. Therefore, their usage can fully test the utility of the proposed feature sampling scheme. If the classifier with features yielded by the multiple sampling scheme is superior to that with previous features for any of these classification algorithms, then, the robustness of the novel features obtained by the multiple sampling scheme is confirmed.

### 2.5. Accuracy Measurement

Ten-fold cross-validation [54–59] was adopted to evaluate the performance of all constructed classifiers. Such method randomly divides the original dataset into ten parts. Each part is singled out one by one as the test set, and the remaining parts constitute the training set. Samples in the test set are predicted by the classifier based on the training set. Thus, each sample is tested exactly once.

For a binary classification problem, four entries can be counted by comparing the predicted and true classes of each sample, that is, true positive (TP), false positive (FP), true negative (TN), and false negative (FN). The following measurements were based on these four entries: sensitivity (SN) (also called recall), specificity (SP), prediction accuracy (ACC), Matthews correlation coefficient (MCC) [20, 21, 37, 60–63], precision, and *F*1-measure. Their definitions are as follows:

(1) $$\begin{array}{c} \text{SN}\left(\text{recall}\right)=\frac{\text{TP}}{\text{TP}+\text{FN}}, \end{array}$$

(2) $$\begin{array}{c} \text{SP}=\frac{\text{TN}}{\text{TN}+\text{FP}}, \end{array}$$

(3) $$\begin{array}{c} \text{ACC}=\frac{\text{TP}+\text{TN}}{\text{TP}+\text{FN}+\text{FP}+\text{TN}}, \end{array}$$

(4) $$\begin{array}{c} \text{MCC}=\frac{\text{TP}\times \text{TN}‐\text{FP}\times \text{FN}}{\sqrt{\left(\text{TN}+\text{FN}\right)\left(\text{TN}+\text{FP}\right)\left(\text{TP}+\text{FN}\right)\left(\text{TP}+\text{FP}\right)}}, \end{array}$$

(5) $$\begin{array}{c} \text{precision}=\frac{\text{TP}}{\text{TP}+\text{FP}}, \end{array}$$

(6) $$\begin{array}{c} F1-\text{measure}=\frac{2\times \text{precision}\times \text{recall}}{\text{precision}+\text{recall}}. \end{array}$$

ACC, MCC, and *F*1-measure use all four entries and thus are more important than the other three measurements. Receiver operating characteristic (ROC) curve [64] and precision-recall (PR) curve were further employed to fully assess the performance of constructed classifiers. These curves indicate the performance of classifiers under different thresholds. ROC curve takes 1-SP as *x*-axis and SN as the *y*-axis, and PR curve takes recall as *x*-axis and precision as *y*-axis. Areas under these two curves (AUROC and AUPR) are important measurements to evaluate the performance of classifiers. Among the abovementioned parameters, MCC was selected as the main measurement.

## 3. Results and Discussion

**Figure 2:**
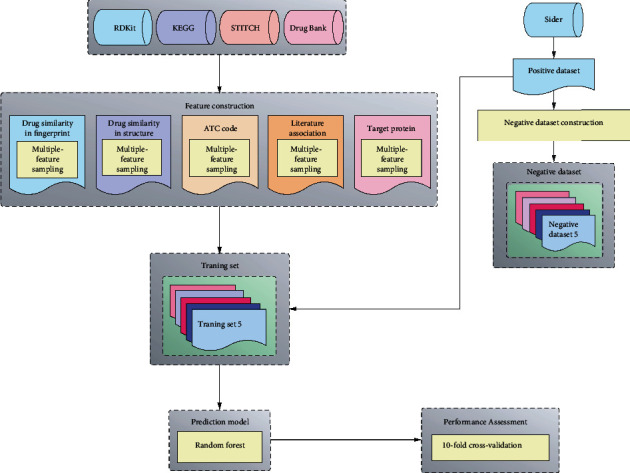
Entire procedures of the method for identification of drug side effects. Positive dataset (reported drug–side effect pairs) is retrieved from SIDER, and five negative datasets are randomly generated. From the four public databases or tools, five drug properties are employed and used to extract features with multiple-feature sampling scheme. Random forest is adopted to build the model and is further evaluated by 10-fold cross-validation.

A novel feature extraction method was proposed to extract essential features from drug–side effect pairs. On the basis of these features, efficient classifiers to predict drug side effects were established. All procedures are illustrated in Figure 2.

### 3.1. Performance of the RF Classifiers with Discrete Strategy

The discrete strategy picks some discrete values in the candidate feature list. Given that the top value in such list is the most important and has been previously selected as the exclusive feature [21, 65], this top value is always picked up as one feature. As mentioned in Section 2.3, the value located at top *q*% place in the list was also selected. In this study, *q* was set as 5, 10, 15, and 20. Values with high ranks in the candidate feature list are more important than those with low ranks, that is, the top value is the most important, followed by values at 5%, 10%, 15%, and 20%. Incremental feature selection was adopted to generate four feature subsets as listed in column 1 of Table 1. With each feature subsets derived from five types of drug associations, a RF classifier was built on each of five datasets and evaluated by 10-fold cross-validation. The average performance is listed in Table 1. MCC followed an increasing trend when the values at top 5%, 10%, 15%, and 20% were added. Other five measurements also generally followed such trend. The RF classifiers with all selected features (top values and those at 5%, 10%, 15%, and 20%) generated the highest MCC of 0.7172. This finding indicated that the features yielded by such multiple-feature sampling scheme were quite efficient for the identification of drug side effects.

**Figure 3:**
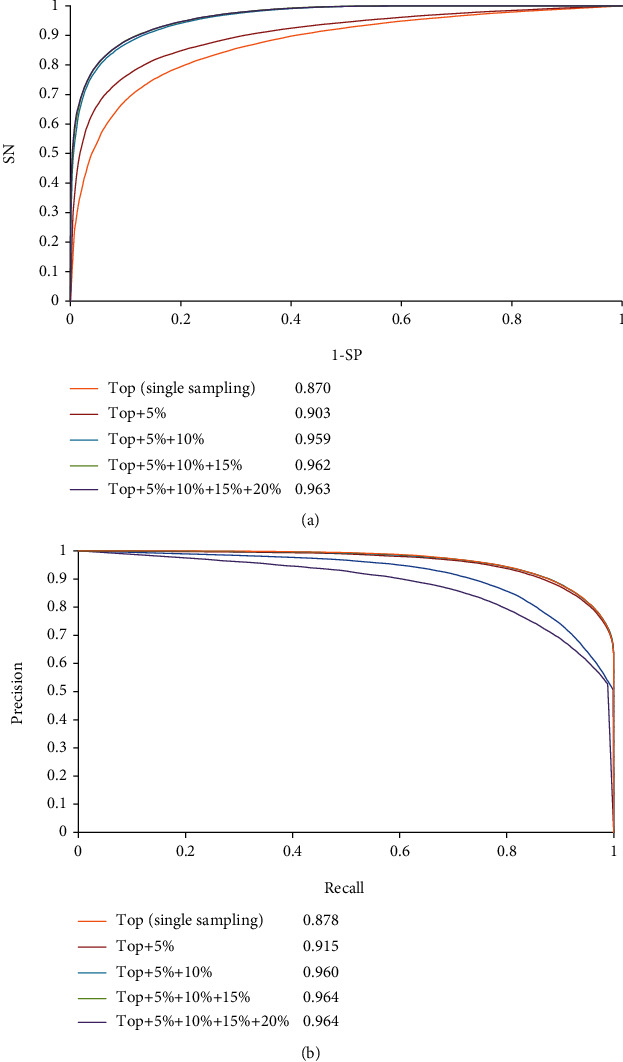
Receiver operating characteristic (ROC) curve and precision-recall (PR) curve of RF classifiers with single-feature sampling scheme and multiple-feature sampling scheme (discrete strategy). (a) ROC curves and (b) PR curves.

The ROC and PR curves of these four RF classifiers were investigated, and the results are shown in Figure 3. All AUROCs and AUPRs were higher than 0.900 and 0.910, respectively, thus, further suggesting the good performance of RF classifiers with discrete strategy.

### 3.2. Performance of RF Classifiers with Continuous Strategy

Different from discrete strategy, continuous strategy selected values from the candidate feature list in a continuous way. As mentioned in Section 2.3, top *q*% values in the candidate feature list can be chosen as features. Here, some *q* values including 10, 20, 30, and 40 and four feature subsets were tested. A RF classifier was also built on each of the five datasets by using the feature subsets derived from the five types of drug associations. Each classifier was assessed by 10-fold cross-validation, and the average performance is listed in Table 2. When *q* = 20 (top 20%), the RF classifier yielded the highest MCC of 0.8661 and generated the ACC of 0.9312, *F*1-measure of 0.9278, SN of 0.8852, SP of 0.9771, and precision of 0.9747. Compared with the RF classifiers with discrete strategy, the best RF with continuous strategy had higher measurements, particularly for MCC (by 15%), ACC (by 7%), and *F*1-measure (by 7%). These results indicated that the features obtained by continuous strategy were more powerful in identifying drug side effects than those yielded by discrete strategy.

**Figure 4:**
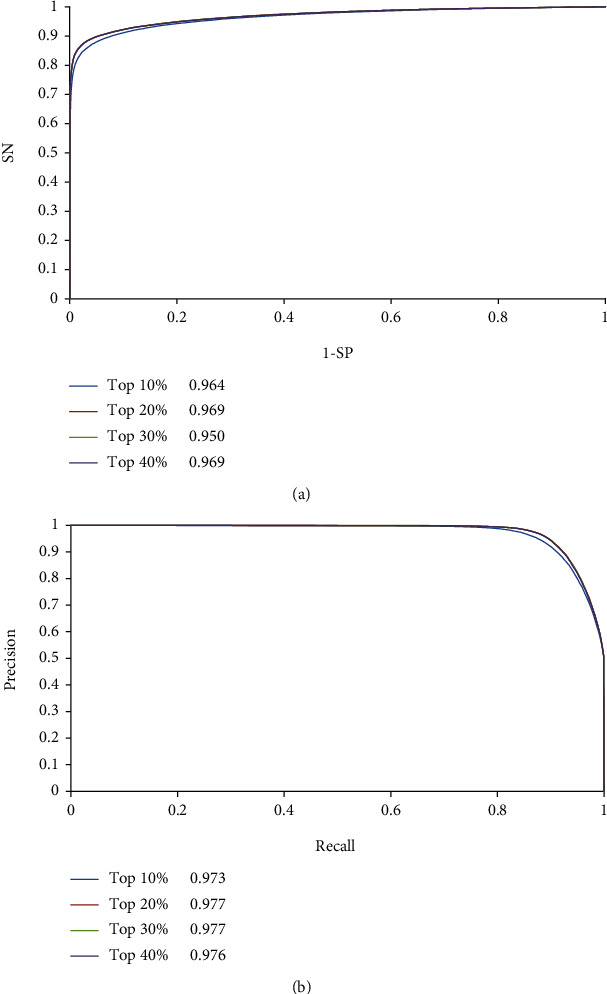
Receiver operating characteristic (ROC) curve and precision-recall (PR) curve of RF classifiers with multiple-feature sampling scheme (continuous strategy). (a) ROC curves and (b) PR curves.

The ROC and PR curves of RF classifiers with continuous strategy were plotted as shown in Figure 4. All ROC curves were close to the point (0, 1), and all PR curves were close to the point (1, 1). The AUROCs and AUPRs were all quite high. Compared with AUROCs and AUPRs for discrete strategy, those for continuous strategy were generally higher. This finding further confirmed that the features yielded by continuous strategy were more powerful than those yielded by discrete strategy.

### 3.3. Comparison of RF Classifiers with Single- and Multiple-Feature Sampling

A multiple-feature sampling scheme was proposed to extract essential features from each drug–side effect pair. Previous studies [21, 22] only picked up the top value as the feature, and this technique was called single sampling scheme. This section compares the RF classifiers with these two feature sampling schemes.

The average performances of RF classifiers with single-feature sampling scheme are listed in Table 3. The MCC was 0.5997, ACC was 0.7999, and *F*1-measure was 0.7988. Other three measurements (SN, SP, and precision) were 0.7948, 0.8049, and 0.8030, respectively. The best performing (highest MCC) RF classifiers with discrete and continuous strategies were selected for comparison and are also listed in Table 3. The MCCs for two strategies were 0.7172 and 0.8661, which were higher than that for the RF classifier with single-feature sampling scheme. Same conclusions can be obtained for other five measurements. The ROC and PR curves of RF classifier with single-feature sampling scheme were also plotted (Figure 3) and were found to be always under those of RF classifiers with discrete strategy. The AUROC and AUPR of the RF classifier with single-feature sampling scheme were 0.870 and 0.878, respectively, which were also lower than those of the RF classifier with discrete strategy. For the RF classifier with continuous strategy, its AUROCs and AUPRs (Figure 4) were even better than those of the RF classifier with discrete strategy and were also higher than those of the RF classifier with single-feature sampling scheme. All these results implied that the features yielded by the multiple sampling scheme contained more essential information of drug–side effect pairs than those obtained by the single sampling scheme. These features provide RF with improved performance.

### 3.4. Performance of Other Classifiers with Multiple-Feature Sampling Scheme

The RF classifiers with features yielded by multiple sampling (discrete strategy) were superior to those with features yielded by single sampling, and the RF classifiers with continuous strategy were better than those with discrete strategy. However, the relevance of this result to the selection of classification algorithms must be explored. In this section, 12 classification algorithms mentioned in Section 2.4 were tested. The classifiers with different algorithms and all feature subsets used for RF were constructed and evaluated by 10-fold cross-validation. The predicted results are listed in Tables S1–S24.

**Figure 5:**
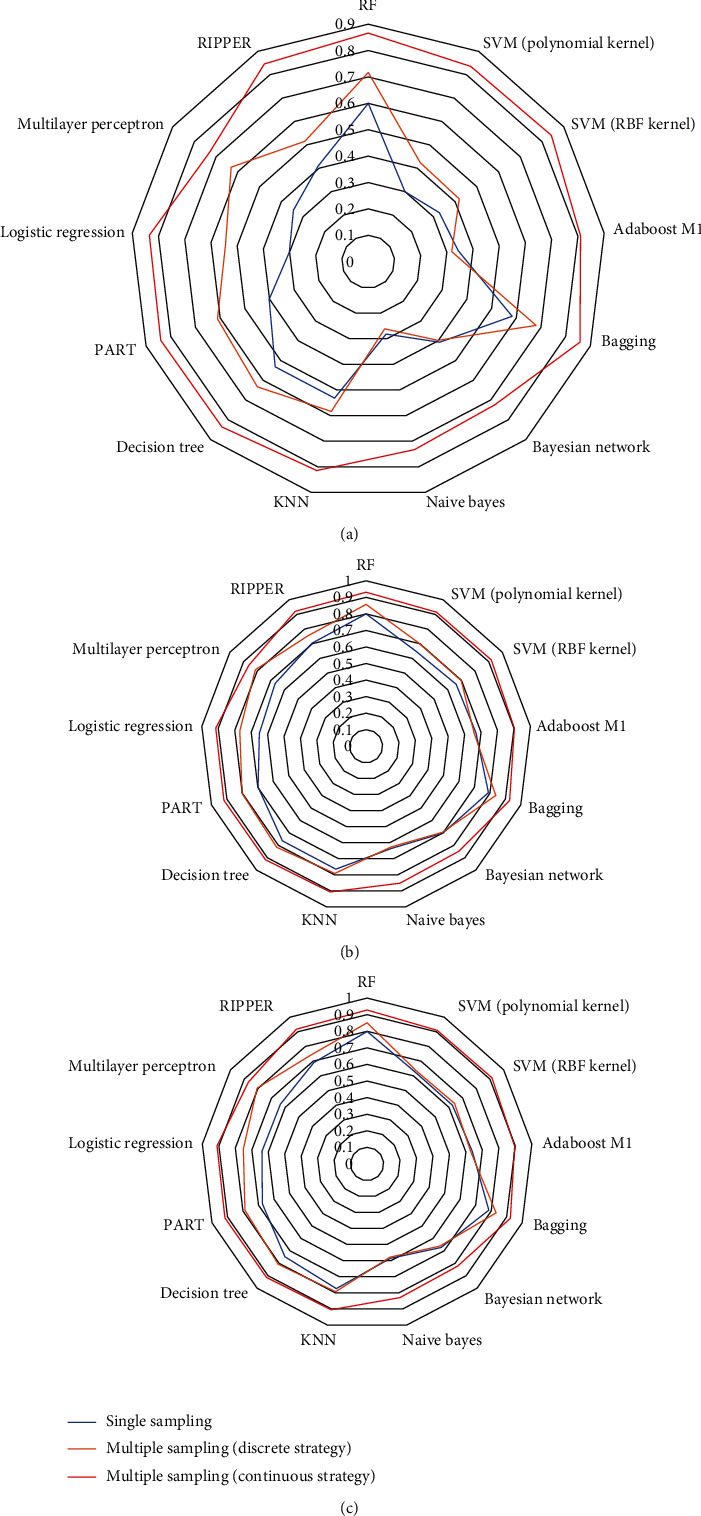
Radar graphs to show performance of classifiers with single- and multiple-feature sampling schemes. (a) MCC; (b) ACC; (c) *F*1-measure. Classifiers with multiple-feature sampling scheme (continuous strategy) provide best performance.

The performances of classifiers with single sampling and the best performance of classifiers with multiple sampling are listed in Table 4. The classifiers with multiple sampling (discrete strategy) were generally better than those with single sampling, and those with continuous strategy were superior to those with discrete strategy and single sampling. For a visualized confirmation, a radar graph was plotted for each value of ACC, MCC, and *F*1-measure as illustrated in Figure 5. For each measurement, the area in the closed curve of classifiers with multiple sampling (continuous strategy) was the largest, followed by the closed curve of classifiers with multiple sampling (discrete strategy); the area in the closed curve of classifiers with single sampling was the smallest. On the basis of these results, multiple sampling scheme is more efficient to capture the essential properties of drug–side effect pairs than single sampling scheme, and continuous strategy is better than discrete strategy.

### 3.5. Analysis of the Parameter of Continuous Strategy

For the continuous strategy, the parameter *q* is a key factor that determines the number of selected features from the candidate feature list. Here, its influence on the performance of classifiers was investigated.

**Figure 6:**
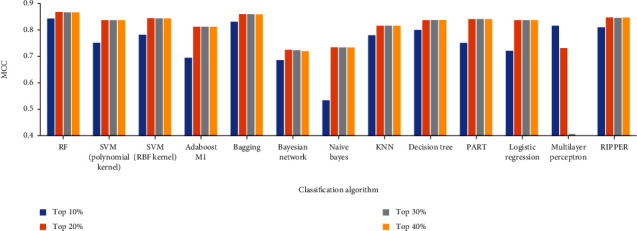
Performance of classifiers with continuous strategy under different parameters.

For RF classifiers, the highest MCC of 0.8661 was achieved when *q* = 20 (Table 2). For other classifiers with different classification algorithms, *q* = 20 always yields the best performance as shown in Figure 6. Among the 13 classifiers with different classification algorithms, 10 provided the best performance when *q* = 20, occupying 76.92%. Meanwhile, two yielded the best performance when *q* = 30. This phenomenon was reasonable. When *q* is extremely small, some essential information of drug–side effect pairs cannot be included. When *q* is large, several noises may be employed. Current investigation revealed that the values of *q* can be taken in an interval [20, 30].

## 4. Conclusions

This study prevents a novel investigation on drug side effects. The contributions contained two aspects. One was the multiple-feature sampling scheme that can extract essential features from drug–side effect pairs, and other one was novel computational methods for the identification of drug side effects based on the features yielded by the multiple sampling scheme. Classifiers were built on the basis of different classification algorithms. By comparison, the classifiers using features yielded by the multiple sampling scheme performed better than those using features yielded by the single sampling scheme. The proposed classifiers can be useful tools to identify drug side effects, and the novel feature extraction scheme can be applied to other similar biological or medical problems.

## Figures and Tables

**Table 1:** Performance of the RF classifiers with discrete strategy.

Feature sampling	SN	SP	ACC	MCC	Precision	*F*1 measure
Top + 5%	0.8072	0.8694	0.8383	0.6780	0.8608	0.8331
Top + 5% + 10%	0.8209	0.8829	0.8519	0.7058	0.8751	0.8467
Top + 5% + 10% + 15%	0.8214	0.8907	0.8561	0.7145	0.8825	0.8505
Top + 5% + 10% + 15% + 20%	0.8201	0.8944	0.8573	0.7172	0.8860	0.8514

**Table 2:** Performance of the RF classifiers with continuous strategy.

Feature sampling	SN	SP	ACC	MCC	Precision	*F*1 measure
Top 10%	0.8737	0.9644	0.9190	0.8416	0.9609	0.9152
Top 20%	0.8852	0.9771	0.9312	0.8661	0.9747	0.9278
Top 30%	0.8844	0.9770	0.9307	0.8652	0.9747	0.9273
Top 40%	0.8834	0.9775	0.9305	0.8648	0.9751	0.9270

**Table 3:** Comparison of RF classifiers with single- and multiple-feature sampling schemes.

Scheme		SN	SP	ACC	MCC	Precision	*F*1 measure
Single sampling		0.7948	0.8049	0.7999	0.5997	0.8030	0.7988
Multiple sampling	Discrete strategy	0.8201	0.8944	0.8573	0.7172	0.8860	0.8514
	Continuous strategy	0.8852	0.9771	0.9312	0.8661	0.9747	0.9278

**Table 4:** Performance of classifiers with different classification algorithms and feature extraction schemes.

Classification algorithm	Feature extraction scheme		ACC	MCC	*F*1-measure
SVM (polynomial kernel)	Single sampling		0.6487	0.2997	0.6252
	Multiple sampling	Discrete strategy	0.6989	0.4240	0.6357
		Continuous strategy	0.9152	0.8356	0.9101

SVM (RBF kernel)	Single sampling		0.6608	0.3276	0.6251
	Multiple sampling	Discrete strategy	0.6987	0.4188	0.6415
		Continuous strategy	0.9191	0.8428	0.9147

Adaboost M1	Single sampling		0.6693	0.3435	0.6392
	Multiple sampling	Discrete strategy	0.6574	0.3186	0.6287
		Continuous strategy	0.9024	0.8102	0.8963

Bagging	Single sampling		0.7909	0.5828	0.7848
	Multiple sampling	Discrete strategy	0.8386	0.6799	0.8317
		Continuous strategy	0.9273	0.8580	0.9240

Bayesian network	Single sampling		0.7007	0.4076	0.6722
	Multiple sampling	Discrete strategy	0.6950	0.3980	0.6614
		Continuous strategy	0.8473	0.7236	0.8225

Naive Bayes	Single sampling		0.6368	0.2822	0.5859
	Multiple sampling	Discrete strategy	0.6272	0.2616	0.5782
		Continuous strategy	0.8528	0.7329	0.8296

KNN	Single sampling		0.7652	0.5321	0.7740
	Multiple sampling	Discrete strategy	0.7918	0.5838	0.7931
		Continuous strategy	0.9071	0.8148	0.9054

Decision tree	Single sampling		0.7635	0.5315	0.7471
	Multiple sampling	Discrete strategy	0.8154	0.6333	0.8080
		Continuous strategy	0.9170	0.8359	0.9142

PART	Single sampling		0.6986	0.4015	0.6753
	Multiple sampling	Discrete strategy	0.8022	0.6105	0.7874
		Continuous strategy	0.9192	0.8402	0.9166

Logistic regression	Single sampling		0.6501	0.3008	0.6383
	Multiple sampling	Discrete strategy	0.7690	0.5442	0.7515
		Continuous strategy	0.9157	0.8353	0.9115

Multilayer perceptron	Single sampling		0.6680	0.3438	0.6352
	Multiple sampling	Discrete strategy	0.8139	0.6305	0.8052
		Continuous strategy	0.8616	0.7299	0.8688

RIPPER	Single sampling		0.7037	0.4090	0.6904
	Multiple sampling	Discrete strategy	0.7546	0.5156	0.7382
		Continuous strategy	0.9215	0.8460	0.9181

## Data Availability

The original data used to support the findings of this study are available at SIDER and in supplementary information files.

## Supplementary Materials

Supplementary Materials Table S1: performance of SVM (polynomial kernel) classifier with discrete strategy. Table S2: performance of SVM (polynomial kernel) classifier with continuous strategy. Table S3: performance of SVM (RBF kernel) classifier with discrete strategy. Table S4: performance of SVM (RBF kernel) classifier with continuous strategy. Table S5: performance of Adaboost M1 classifier with discrete strategy. Table S6: performance of Adaboost M1 classifier with continuous strategy. Table S7: performance of Bagging classifier with discrete strategy. Table S8: performance of Bagging classifier with continuous strategy. Table S9: performance of Bayesian network classifier with discrete strategy. Table S10: performance of Bayesian network classifier with continuous strategy. Table S11: performance of Naive Bayes classifier with discrete strategy. Table S12: performance of Naive Bayes classifier with continuous strategy. Table S13: performance of KNN classifier with discrete strategy. Table S14: performance of KNN classifier with continuous strategy. Table S15: performance of decision tree classifier with discrete strategy. Table S16: performance of decision tree classifier with continuous strategy. Table S17: performance of PART classifier with discrete strategy. Table S18: performance of PART classifier with continuous strategy. Table S19: performance of logistic regression classifier with discrete strategy. Table S20: performance of logistic regression classifier with continuous strategy. Table S2: performance of multilayer perceptron classifier with discrete strategy. Table S22: performance of multilayer perceptron classifier with continuous strategy. Table S23: performance of RIPPER classifier with discrete strategy. Table S24: performance of RIPPER classifier with continuous strategy.
