# Supplementary material for: Similarity-Based Method with Multiple-Feature Sampling for Predicting Drug Side Effects
Source: Comput Math Methods Med. 2022 Apr 1;2022:9547317. doi: 10.1155/2022/9547317 (PMC8993545; doi:10.1155/2022/9547317)
Supplement: Supplementary Materials — Table S1: performance of SVM (polynomial kernel) classifier with discrete strategy. Table S2: performance of SVM (polynomial kernel) classifier with continuous strategy. Table S3: performance of SVM (RBF kernel) classifier with discrete strategy. Table S4: performance of SVM (RBF kernel) classifier with continuous strategy. Table S5: performance of Adaboost M1 classifier with discrete strategy. Table S6: performance of Adaboost M1 classifier with continuous strategy. Table S7: performance of Bagging classifier with discrete strategy. Table S8: performance of Bagging classifier with continuous strategy. Table S9: performance of Bayesian network classifier with discrete strategy. Table S10: performance of Bayesian network classifier with continuous strategy. Table S11: performance of Naive Bayes classifier with discrete strategy. Table S12: performance of Naive Bayes classifier with continuous strategy. Table S13: performance of KNN classifier with discrete strategy. Table S14: performance of KNN classifier with continuous strategy. Table S15: performance of decision tree classifier with discrete strategy. Table S16: performance of decision tree classifier with continuous strategy. Table S17: performance of PART classifier with discrete strategy. Table S18: performance of PART classifier with continuous strategy. Table S19: performance of logistic regression classifier with discrete strategy. Table S20: performance of logistic regression classifier with continuous strategy. Table S2: performance of multilayer perceptron classifier with discrete strategy. Table S22: performance of multilayer perceptron classifier with continuous strategy. Table S23: performance of RIPPER classifier with discrete strategy. Table S24: performance of RIPPER classifier with continuous strategy. [file 9547317.f1.pdf]

Table S1. Performance of SVM (polynomial kernel) classifier with discrete strategy.

Feature sampling	SN	SP	ACC	MCC	Precision	F1 measure
Top (single sampling)	0.5860	0.7113	0.6487	0.2997	0.6700	0.6252
Top+5%	0.4956	0.8685	0.6821	0.3924	0.7903	0.6092
Top+5%+10%	0.5095	0.8775	0.6935	0.4162	0.8061	0.6242
Top+5%+10%+15%	0.5210	0.8747	0.6979	0.4230	0.8059	0.6328
Top+5%+10%+15%+20%	0.5257	0.8721	0.6989	0.4240	0.8041	0.6357

Table S2. Performance of SVM (polynomial kernel) classifier with continuous strategy.

Feature sampling	SN	SP	ACC	MCC	Precision	F1 measure
Top 10%	0.8446	0.9037	0.8742	0.7496	0.8977	0.8703
Top 20%	0.8606	0.9698	0.9152	0.8356	0.9661	0.9101
Top 30%	0.8606	0.9698	0.9152	0.8356	0.9661	0.9101
Top 40%	0.8606	0.9698	0.9152	0.8355	0.9661	0.9101

Table S3. Performance of SVM (RBF kernel) classifier with discrete strategy.

Feature sampling	SN	SP	ACC	MCC	Precision	F1 measure
Top (single sampling)	0.5656	0.7560	0.6608	0.3276	0.6986	0.6251
Top+5%	0.5181	0.8225	0.6703	0.3576	0.7448	0.6111
Top+5%+10%	0.5288	0.8488	0.6888	0.3984	0.7770	0.6290
Top+5%+10%+15%	0.5406	0.8569	0.6987	0.4188	0.7899	0.6415
Top+5%+10%+15%+20%	0.5228	0.8555	0.6892	0.4012	0.7835	0.6271

Table S4. Performance of SVM (RBF kernel) classifier with continuous strategy.

Feature sampling	SN	SP	ACC	MCC	Precision	F1 measure
Top 10%	0.8263	0.9483	0.8873	0.7804	0.9411	0.8800
Top 20%	0.8679	0.9703	0.9191	0.8428	0.9669	0.9147
Top 30%	0.8679	0.9703	0.9191	0.8427	0.9669	0.9147
Top 40%	0.8679	0.9703	0.9191	0.8427	0.9669	0.9147

Table S5. Performance of Adaboost M1 classifier with discrete strategy.

Feature sampling	SN	SP	ACC	MCC	Precision	F1 measure
Top (single sampling)	0.5860	0.7526	0.6693	0.3435	0.7032	0.6392
Top+5%	0.5801	0.7347	0.6574	0.3186	0.6863	0.6287
Top+5%+10%	0.5768	0.7356	0.6562	0.3165	0.6858	0.6266
Top+5%+10%+15%	0.5809	0.7278	0.6544	0.3121	0.6809	0.6270
Top+5%+10%+15%+20%	0.5809	0.7278	0.6544	0.3121	0.6809	0.6270

Table S6. Performance of Adaboost M1 classifier with continuous strategy.

Feature sampling	SN	SP	ACC	MCC	Precision	F1 measure
Top 10%	0.6993	0.9677	0.8335	0.6932	0.9567	0.8068
Top 20%	0.8466	0.9581	0.9024	0.8102	0.9529	0.8963
Top 30%	0.8466	0.9581	0.9024	0.8102	0.9529	0.8963
Top 40%	0.8466	0.9581	0.9024	0.8102	0.9529	0.8963

Table S7. Performance of Bagging classifier with discrete strategy.

Feature sampling	SN	SP	ACC	MCC	Precision	F1 measure
Top (single sampling)	0.7626	0.8192	0.7909	0.5828	0.8084	0.7848
Top+5%	0.7908	0.8703	0.8305	0.6632	0.8591	0.8235
Top+5%+10%	0.8006	0.8725	0.8366	0.6757	0.8626	0.8299
Top+5%+10%+15%	0.8012	0.8759	0.8386	0.6799	0.8659	0.8317
Top+5%+10%+15%+20%	0.8018	0.8749	0.8384	0.6793	0.8650	0.8316

Table S8. Performance of Bagging classifier with continuous strategy.

Feature sampling	SN	SP	ACC	MCC	Precision	F1 measure
Top 10%	0.8707	0.9561	0.9134	0.8298	0.9520	0.9095
Top 20%	0.8845	0.9702	0.9273	0.8580	0.9674	0.9240
Top 30%	0.8845	0.9702	0.9273	0.8580	0.9674	0.9240
Top 40%	0.8843	0.9701	0.9272	0.8576	0.9673	0.9238

Table S9. Performance of Bayesian network classifier with discrete strategy.

Feature sampling	SN	SP	ACC	MCC	Precision	F1 measure
Top (single sampling)	0.6137	0.7877	0.7007	0.4076	0.7430	0.6722
Top+5%	0.5959	0.7941	0.6950	0.3980	0.7433	0.6614
Top+5%+10%	0.5879	0.7987	0.6933	0.3955	0.7449	0.6571
Top+5%+10%+15%	0.5838	0.7995	0.6916	0.3925	0.7443	0.6543
Top+5%+10%+15%+20%	0.5819	0.7965	0.6892	0.3875	0.7409	0.6518

Table S10. Performance of Bayesian network classifier with continuous strategy.

Feature sampling	SN	SP	ACC	MCC	Precision	F1 measure
Top 10%	0.7433	0.9291	0.8362	0.6844	0.9130	0.8194
Top 20%	0.7076	0.9871	0.8473	0.7236	0.9821	0.8225
Top 30%	0.6911	0.9959	0.8435	0.7214	0.9941	0.8153
Top 40%	0.6847	0.9977	0.8412	0.7186	0.9967	0.8117

Table S11. Performance of Naive Bayes classifier with discrete strategy.

Feature sampling	SN	SP	ACC	MCC	Precision	F1 measure
Top (single sampling)	0.5138	0.7597	0.6368	0.2822	0.6814	0.5859
Top+5%	0.5111	0.7433	0.6272	0.2616	0.6657	0.5782
Top+5%+10%	0.5016	0.7499	0.6257	0.2596	0.6673	0.5727
Top+5%+10%+15%	0.4875	0.7612	0.6244	0.2586	0.6713	0.5648
Top+5%+10%+15%+20%	0.4701	0.7710	0.6205	0.2528	0.6725	0.5534

Table S12. Performance of Naive Bayes classifier with continuous strategy.

Feature sampling	SN	SP	ACC	MCC	Precision	F1 measure
Top 10%	0.5311	0.9518	0.7415	0.5323	0.9168	0.6726
Top 20%	0.7173	0.9882	0.8528	0.7329	0.9837	0.8296
Top 30%	0.7127	0.9906	0.8516	0.7322	0.9869	0.8276
Top 40%	0.7122	0.9907	0.8514	0.7319	0.9871	0.8273

Table S13. Performance of KNN classifier with discrete strategy.

Feature sampling	SN	SP	ACC	MCC	Precision	F1 measure
Top (single sampling)	0.8039	0.7266	0.7652	0.5321	0.7462	0.7740
Top+5%	0.7776	0.7666	0.7721	0.5443	0.7692	0.7734
Top+5%+10%	0.7878	0.7870	0.7874	0.5749	0.7867	0.7871
Top+5%+10%+15%	0.7933	0.7863	0.7898	0.5797	0.7875	0.7903
Top+5%+10%+15%+20%	0.7987	0.7850	0.7918	0.5838	0.7876	0.7931

Table S14. Performance of KNN classifier with continuous strategy.

Feature sampling	SN	SP	ACC	MCC	Precision	F1 measure
Top 10%	0.8753	0.9027	0.8890	0.7782	0.9000	0.8874
Top 20%	0.8890	0.9253	0.9071	0.8148	0.9224	0.9054
Top 30%	0.8890	0.9253	0.9071	0.8148	0.9224	0.9054
Top 40%	0.8890	0.9253	0.9071	0.8148	0.9224	0.9054

Table S15. Performance of decision tree classifier with discrete strategy.

Feature sampling	SN	SP	ACC	MCC	Precision	F1 measure
Top (single sampling)	0.6988	0.8282	0.7635	0.5315	0.8027	0.7471
Top+5%	0.7479	0.8529	0.8004	0.6041	0.8357	0.7893
Top+5%+10%	0.7684	0.8547	0.8116	0.6259	0.8407	0.8025
Top+5%+10%+15%	0.7709	0.8549	0.8129	0.6284	0.8413	0.8042
Top+5%+10%+15%+20%	0.7825	0.8483	0.8154	0.6333	0.8372	0.8080

Table S16. Performance of decision tree classifier with continuous strategy.

Feature sampling	SN	SP	ACC	MCC	Precision	F1 measure
Top 10%	0.8686	0.9285	0.8986	0.7986	0.9241	0.8955
Top 20%	0.8847	0.9492	0.9169	0.8357	0.9458	0.9141
Top 30%	0.8848	0.9492	0.9170	0.8359	0.9458	0.9142
Top 40%	0.8848	0.9492	0.9170	0.8359	0.9458	0.9142

Table S17. Performance of PART classifier with discrete strategy.

Feature sampling	SN	SP	ACC	MCC	Precision	F1 measure
Top (single sampling)	0.6272	0.7700	0.6986	0.4015	0.7320	0.6753
Top+5%	0.6986	0.8395	0.7690	0.5442	0.8143	0.7515
Top+5%+10%	0.7473	0.8366	0.7920	0.5882	0.8214	0.7810
Top+5%+10%+15%	0.7526	0.8443	0.7985	0.5996	0.8285	0.7886
Top+5%+10%+15%+20%	0.7351	0.8694	0.8022	0.6105	0.8492	0.7874

Table S18. Performance of PART classifier with continuous strategy.

Feature sampling	SN	SP	ACC	MCC	Precision	F1 measure
Top 10%	0.8163	0.9272	0.8718	0.7491	0.9157	0.8615
Top 20%	0.8864	0.9515	0.9190	0.8399	0.9482	0.9162
Top 30%	0.8883	0.9502	0.9192	0.8402	0.9469	0.9166
Top 40%	0.8866	0.9515	0.9190	0.8399	0.9481	0.9163

Table S19. Performance of logistic regression classifier with discrete strategy.

Feature sampling	SN	SP	ACC	MCC	Precision	F1 measure
Top (single sampling)	0.6174	0.6828	0.6501	0.3008	0.6606	0.6383
Top+5%	0.6986	0.8395	0.7690	0.5442	0.8143	0.7515
Top+5%+10%	0.5886	0.8210	0.7048	0.4209	0.7657	0.6654
Top+5%+10%+15%	0.5698	0.8156	0.6927	0.3976	0.7552	0.6495
Top+5%+10%+15%+20%	0.6321	0.8432	0.7377	0.4859	0.7990	0.7055

Table S20. Performance of logistic regression classifier with continuous strategy.

Feature sampling	SN	SP	ACC	MCC	Precision	F1 measure
Top 10%	0.8505	0.8692	0.8599	0.7198	0.8667	0.8585
Top 20%	0.8687	0.9628	0.9157	0.8353	0.9588	0.9115
Top 30%	0.8687	0.9628	0.9157	0.8353	0.9588	0.9115
Top 40%	0.8687	0.9628	0.9157	0.8353	0.9588	0.9115

Table S21. Performance of multilayer perceptron classifier with discrete strategy.

Feature sampling	SN	SP	ACC	MCC	Precision	F1 measure
Top (single sampling)	0.5732	0.7643	0.6680	0.3438	0.7127	0.6352
Top+5%	0.7465	0.7967	0.7716	0.5450	0.7874	0.7655
Top+5%+10%	0.7560	0.8563	0.8062	0.6160	0.8404	0.7955
Top+5%+10%+15%	0.7715	0.8564	0.8139	0.6305	0.8429	0.8052
Top+5%+10%+15%+20%	0.7731	0.8524	0.8127	0.6280	0.8397	0.8046

Table S22. Performance of multilayer perceptron classifier with continuous strategy.

Feature sampling	SN	SP	ACC	MCC	Precision	F1 measure
Top 10%	0.8565	0.9540	0.9052	0.8144	0.9490	0.9004
Top 20%	0.8948	0.8284	0.8616	0.7299	0.8503	0.8688
Top 30%	0.9528	0.3835	0.6681	0.4058	0.6150	0.7448
Top 40%	0.9999	0.0000	0.5000	-0.0052	0.5000	0.6666

Table S23. Performance of RIPPER classifier with discrete strategy.

Feature sampling	SN	SP	ACC	MCC	Precision	F1 measure
Top (single sampling)	0.6606	0.7468	0.7037	0.4090	0.7231	0.6904
Top+5%	0.6325	0.8086	0.7205	0.4481	0.7676	0.6934
Top+5%+10%	0.6799	0.8106	0.7452	0.4979	0.7819	0.7242
Top+5%+10%+15%	0.6644	0.8110	0.7377	0.4810	0.7794	0.7170
Top+5%+10%+15%+20%	0.7015	0.8078	0.7546	0.5156	0.7852	0.7382

Table S24. Performance of RIPPER classifier with continuous strategy.

Feature sampling	SN	SP	ACC	MCC	Precision	F1 measure
Top 10%	0.8535	0.9509	0.9022	0.8083	0.9456	0.8972
Top 20%	0.8806	0.9625	0.9215	0.8460	0.9591	0.9181
Top 30%	0.8632	0.9753	0.9192	0.8440	0.9722	0.9143
Top 40%	0.8792	0.9631	0.9212	0.8454	0.9597	0.9176
